# Editorial: Advances in multimodal learning: pedagogies, technologies, and analytics

**DOI:** 10.3389/fpsyg.2023.1286092

**Published:** 2023-10-31

**Authors:** Heng Luo

**Affiliations:** Faculty of Artificial Intelligence in Education, Central China Normal University, Wuhan, China

**Keywords:** multimodal learning, multimodal stimuli, multimodal learning spaces, multimodal behaviors, multimodal learning analytics

The rapid development of digital technologies and data-driven techniques has led to advances in multimodal learning featured by multimodality in instructional stimuli, learning spaces, behavioral pattern, and data sources (Blikstein and Worsley, 2016; Di Mitri et al., 2018; Chango et al., 2021). The combination of multiple sensory stimuli (e.g., visual, audio, verbal, tactile, and olfactory) in instructional content is known to promote cognitive performance, sense of presence, and learning engagement (Moreno and Mayer, 1999; Marucci et al., 2021). The fusion of traditional and digital learning spaces enabled by online platforms and extended reality has created more personal, accessible, and risk-free learning conditions that may otherwise be unavailable (Garrison and Kanuka, 2004; Allcoat et al., 2021; Luo et al., 2021). Multimodality in learning also emphasizes the multiplicity of mode of behaviors such as communication, interaction, and regulation, leading to increased interests in their impacts on learning achievement and experience (Mangaroska et al., 2020; Cloude et al., 2022; Ninaus and Sailer, 2022). More importantly, Multimodal Learning Analytics (MMLA) allows traces of various cognitive, behavioral, and affective indicators to be extracted from multiple data sources (e.g., eye-tracking, wearable cameras, gesture recognition systems, infrared imaging, biosensors) to assist the measurement and understanding of complex learning processes (Blikstein and Worsley, 2016; Di Mitri et al., 2018; Chango et al., 2021).

Consequently, to capture the unique benefits of multimodal learning, research attention needs to be paid to four key aspects of multimodality, including learning content, learning space, learning process, and learning analytics. This Research Topic is an attempt to address such a research need by illustrating recent research development of multimodal learning in the four aspects. It comprises a total of 15 articles contributed by 63 authors globally, from academic and research institutions in Mainland China, Taiwan, United States, United Kingdom, Austria, Canada, Spain, and Colombia. Based on the four aspects of multimodality in learning, we classified the 15 articles into four themes: *design of multimodal stimuli, affordances of multimodal learning space, analysis of multimodal behaviors*, and *application of multimodal analytics*.

The first theme addresses the importance of using multimodal stimuli for creating effective instructional content. Mou et al. describe and evaluate a novel instructional media featuring two modes of stimuli: video and in-video text messages in the forms of bullet comments. While this multiplicity of media content increases parasocial interactions among learners, it also induces greater cognitive load and thus hinders learning performance, revealing a potential caveat of using multimodal stimulus in instruction. Xia et al. take a similar interest in instructional video and its impact on motor skill learning and find that the creation and sharing of video lead to increased intrinsic motivation and motor task perseverance, as compared to self-exercise. Han et al. further investigate two specific stimuli (i.e., text annotations and color changes) as visual signals in immersive virtual reality learning environments, and report empirical evidence supporting their effectiveness on learning for students with low prior knowledge levels. The findings of these three studies contribute to the existing literature of multimodal stimuli by exploring new a design feature, learning domain, and learning environment.

The second theme focuses on the fusion of multiple learning spaces and their unique learning affordances. Sbaffi and Zhao document the design and implementation of an online gamified learning module as a hybrid learning space to teach knowledge of academic integrity for university students. Likewise, Zhang et al. explore in-service teachers' professional development in online learning space by designing and validating an instrument to measure the quality of supporting platforms. Zhao and Xue take a special interest in the transition between online and offline learning spaces in the post-pandemic era, highlighting the transitioning challenges in administration, infrastructure, pedagogy, and finance. In addition to online learning space, two studies focus on extended reality (XR) spaces by synthesizing the existing research findings: Amores-Valencia et al. conduct a systematic review of augmented reality in secondary education, and Chen et al. perform a meta-analysis to determine the effect of XR on language learning. Both studies report positive findings regarding the learning effect of XR learning spaces. Lastly, Schmidthaler et al. introduce a game space (Poly-Universe) absent of digital technologies and prove its effectiveness in teaching computational thinking for children in an interdisciplinary fashion.

The third theme explores the patterns of multimodal behaviors in diverse learning contexts using advanced analysis methods. Tlili et al. use a lag sequential analysis approach to examine the impact of personalities on students' navigational patterns among 12 learning behaviors in an online course, and identify the traits of extraversion, conscientiousness, neuroticism, and openness as potential moderating factors. In a similar online learning context, Luo et al. focus on students' multitasking behaviors in online learning. Using structural equation modeling (SEM), the study reveals predictive path relationships among polychronicity, multitasking behavior, and perceived learning performance. Tao et al. shift their research attention to teachers and employ Partial Least Squares to explore the factors affecting teachers' precision teaching ability with a focus on the mediating effect of data consciousness.

The fourth theme highlights the role of MMLA in assisting people to understand and optimize the learning process and environment with data-driven decisions. Wang et al. conduct an MMLA model enabled by natural language processing and machine learning to predict college students' problem-based learning performance in a blended course, informed by both clickstream data and learner-generated text content. Xiao et al. further integrate physiological data such as brainwaves, eye movements, and facial expressions in their MMLA model to predict in-service teachers' engagement and performance in an online training program. Moreover, Li et al. innovatively utilize heart rate to profile synchronized physiological arousal during collaborative argumentation and explore the potential influencing factors such as types of challenge and social regulation focus. Consistent with the previous literature (Blikstein and Worsley, 2016; Emerson et al., 2020), the three contributions demonstrate the superiority of multimodal data over omni-modal data in predicting learning performance and experience.

In conclusion, the current Research Topic presents recent findings regarding four important aspects of multimodal learning. It is our hope that the contributions of this topic can extend our conceptual, practical, and methodological understanding of learning in the digital era, and lead to a greater breadth of research perspectives in this rapidly evolving field.

## Author contributions

HL: Writing—original draft, Writing—review & editing.
